# Investigating the Mechanisms of Action of Depside Salt from* Salvia miltiorrhiza* Using Bioinformatic Analysis

**DOI:** 10.1155/2017/5671860

**Published:** 2017-11-19

**Authors:** Hua Li, Hongying Liu

**Affiliations:** ^1^Shanghai Institute of Cardiovascular Diseases, Zhongshan Hospital, Fudan University, Shanghai 200032, China; ^2^Institutes of Biomedical Sciences, Fudan University, Shanghai 200032, China; ^3^Institutes of Panvascular Medicine, Fudan University, Shanghai 200032, China; ^4^Shanghai Green Valley Pharmaceutical Company, Shanghai 201203, China

## Abstract

*Salvia miltiorrhiza* is a traditional Chinese medicinal herb used for treating cardiovascular diseases. Depside salt from* S. miltiorrhiza* (DSSM) contains the following active components: magnesium lithospermate B, lithospermic acid, and rosmarinic acid. This study aimed to reveal the mechanisms of action of DSSM. After searching for DSSM-associated genes in GeneCards, Search Tool for Interacting Chemicals, SuperTarget, PubChem, and Comparative Toxicogenomics Database, they were subjected to enrichment analysis using Multifaceted Analysis Tool for Human Transcriptome. A protein-protein interaction (PPI) network was visualised; module analysis was conducted using the Cytoscape software. Finally, a transcriptional regulatory network was constructed using the TRRUST database and Cytoscape. Seventy-three DSSM-associated genes were identified. JUN, TNF, NFKB1, and FOS were hub nodes in the PPI network. Modules 1 and 2 were identified from the PPI network, with pathway enrichment analysis, showing that the presence of* NFKB1* and* BCL2* in module 1 was indicative of a particular association with the NF-*κ*B signalling pathway. JUN, TNF, NFKB1, FOS, and BCL2 exhibited notable interactions among themselves in the PPI network. Several regulatory relationships (such as* JUN *→* TNF/FOS*,* FOS *→* NFKB1* and* NFKB1 *→* BCL2/TNF*) were also found in the regulatory network. Thus, DSSM exerts effects against cardiovascular diseases by targeting* JUN*,* TNF*,* NFKB1*,* FOS*, and* BCL2*.

## 1. Introduction

*Salvia miltiorrhiza* (also named Danshen) is widely used as a traditional Chinese medicinal herb to prevent and treat vascular diseases [1, 2]. Depside salt from* S. miltiorrhiza* (DSSM) is a new medicine that contains the active components magnesium lithospermate B (MLB), lithospermic acid (LA), and rosmarinic acid (RA) [3]. MLB plays protective roles in relieving atherosclerosis and combating myocardial ischaemia-reperfusion injury [4]. RA and LA also have beneficial effects on cardiovascular diseases, such as atherosclerosis and neointimal hyperplasia [5, 6]. Thus, understanding the mechanisms of action of DSSM is important for its better utilisation in a clinical setting.

In patients with unstable angina, DSSM can suppress platelet activation and aggregation as well as matrix metallopeptidase 9* (MMP-9)* expression and secretion [7]. MLB protects against diabetic atherosclerosis by inducing the nuclear factor erythroid 2-related factor-2-antioxidant responsive element-NAD(P)H: quinone oxidoreductase-1 pathway [8]. Du et al. reported that MLB can be used to treat ischaemic heart diseases as it specifically inhibits transforming growth factor *β*-activated protein kinase 1-binding protein 1-p38 apoptosis signalling [9]. Kim et al. also demonstrated that RA inhibits adriamycin-induced cardiotoxicity by suppressing reactive oxygen species generation as well as extracellular signal-regulated kinase and c-Jun N-terminal kinase (JNK) activation [10]. Moreover, LA inhibits foetal bovine serum-induced vascular smooth muscle cell (VSMC) proliferation by arresting cell cycle progression and suppressing cyclin D1 expression and lipopolysaccharide-induced VSMC migration by downregulating* MMP-9* expression; thus, LA may be used for preventing neointimal hyperplasia, restenosis, and atherosclerosis [11]. However, no comprehensive survey of DSSM-associated genes has been reported.

In this study, we searched for DSSM-associated genes in several common databases. Subsequently, we applied multiple bioinformatic methods to identify further DSSM-associated key genes; these methods included enrichment analysis, protein-protein interaction (PPI) network and module analyses, and transcriptional regulatory network analysis. This study may provide a better understanding of the mechanisms of action of DSSM.

## 2. Methods

### 2.1. Search for DSSM-Associated Genes

Several databases were used in this study. The human genomic database GeneCards (http://www.genecards.org/) was used [12], which provides concise genome, transcriptome, proteome, and function data of all predicted and known human genes. Search Tool for Interacting Chemicals (STICH, version 5.0, http://stitch.embl.de/) was also sued [13], which is a database that integrates the interactions between chemicals and proteins. SuperTarget (http://insilico.charite.de/supertarget/) [14] was also used, which is a database that includes drug-associated information correlated with drug metabolism, adverse drug effects, medical indications, Gene Ontology (GO) terms, and pathways for target proteins. The PubChem (https://www.ncbi.nlm.nih.gov/pccompound/) [15] database was also used, which provides information on chemical substances and corresponding biological activities and is linked to the National Institutes of Health PubMed Entrez. Moreover, the study features the Comparative Toxicogenomics Database (CTD, http://ctdbase.org/) [16], which is a database associated with environmental chemical-gene product interactions as well as the transportation and accumulation of chemical substances in the human body. In GeneCards [12], STICH (parameters set as organism = human, score > 0.4) [13], SuperTarget [14], PubChem [15], and CTD [16] databases, “magnesium lithospermate B”, “lithospermic acid” and “rosmarinic acid” were used as keywords to search for genes associated with them (which were combined into a set of DSSM-associated genes).

### 2.2. Functional and Pathway Enrichment Analyses

The GO (http://www.geneontology.org) database describes the associations of gene products with the categories of biological process (BP), molecular function (MF), and cellular component (CC), as well as with more specific subcategories [17]. The Kyoto Encyclopedia of Genes and Genomes (KEGG, http://www.genome.ad.jp/kegg) is a database used for annotating the functions of genes or other molecules [18]. The “BioCloud” online platform was developed for managing problems encountered in the analysis of high-throughput data. By applying the Multifaceted Analysis Tool for Human Transcriptome (MATHT, http://www.biocloudservice.com) in the “BioCloud” online platform, GO functional and KEGG pathway enrichment analyses were conducted for DSSM-associated genes, setting a threshold of <0.05 for the false discovery rate (FDR).

### 2.3. PPI Network and Module Analyses

On combining the Search Tool for the Retrieval of Interacting Genes (STRING, version 10.0, http://www.string-db.org/, combined score > 0.4) [19], Biological General Repository for Interaction Datasets (BioGRID, version 3.4, https://wiki.thebiogrid.org/) [20] and Human Protein Reference Database (release 9, http://www.hprd.org/) [21] interaction databases, PPI pairs among DSSM-associated genes were predicted. Subsequently, the PPI network was visualised for DSSM-associated genes using the Cytoscape software (http://www.cytoscape.org) [22]. Using the CytoNCA plug-in [23] (version 2.1.6, http://apps.cytoscape.org/apps/cytonca) in Cytoscape, degree centrality (DC), betweenness centrality (BC), and closeness centrality of the nodes were analysed to obtain the hub proteins in the PPI network [24]. The parameter was set as “without weight.”

Based on the MCODE plug-in [25] (version 1.4.2; http://apps.cytoscape.org/apps/mcode; parameters set as degree cut-off = 2, maximum depth = 100, node score cut-off = 0.2, and *K*-core = 2) in Cytoscape, module analysis was conducted for the PPI network. Subsequently, KEGG pathway enrichment analysis was performed for the nodes of significant modules, with FDR < 0.05 as the cut-off criterion.

### 2.4. Transcriptional Regulatory Network Construction

Using the transcriptional regulatory relationships unravelled by a sentence-based text-mining (TRRUST, http://www.grnpedia.org/trrust/) [26] database, transcription factors (TFs) among DSSM-associated genes were searched and then their targets were screened. Finally, a transcriptional regulatory network was constructed using Cytoscape [22].

## 3. Results

### 3.1. Search for DSSM-Associated Genes

The numbers of MLB-, RA-, and LA-associated genes identified from GeneCards, STICH, SuperTarget, PubChem, and CTD databases are listed in Table 1. The MLB-, RA-, and LA-associated genes were combined into a group of 73 DSSM-associated genes.

### 3.2. Functional and Pathway Enrichment Analyses

**Figure 1:**
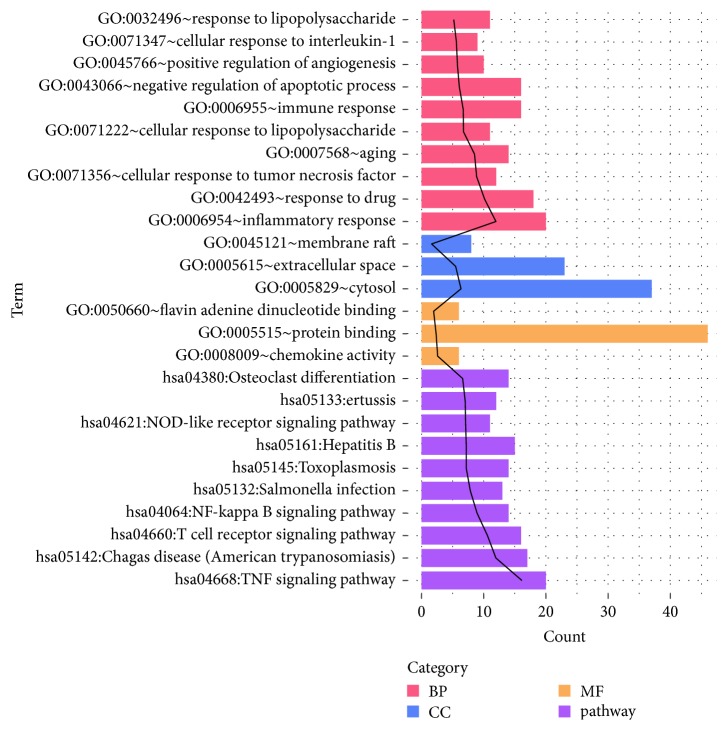
The main BP, CC, and MF terms as well as KEGG pathways enriched for DSSM-associated genes. BP, biological process; CC, cellular component; MF, molecular function; KEGG, Kyoto Encyclopedia of Genes and Genomes; DSSM, depside salt from* Salvia miltiorrhiza*.

The main BP, CC, and MF terms, as well as KEGG pathways enriched for DSSM-associated genes, are shown in Figure 1. The significantly enriched terms included inflammatory response (BP), cytosol (CC), protein binding (MF), and tumour necrosis factor (TNF) signalling pathway (KEGG pathway).

### 3.3. PPI Network and Module Analyses

**Figure 2:**
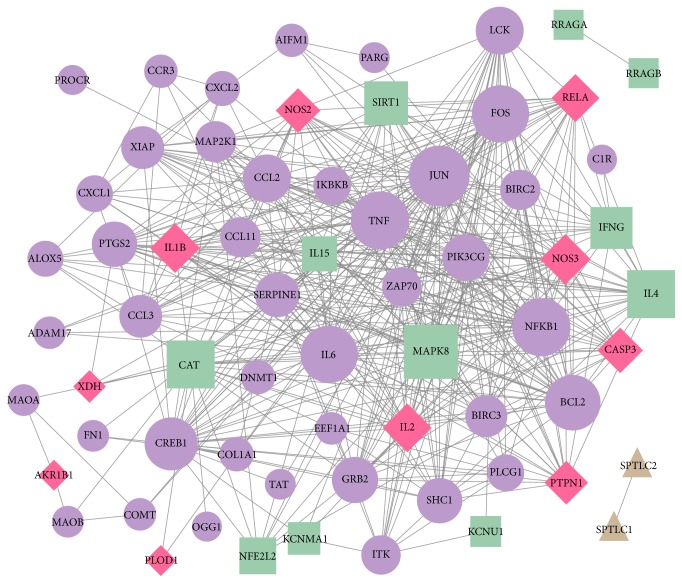
The protein-protein interaction (PPI) network constructed for the depside salt from* Salvia miltiorrhiza*-associated genes. Green squares, purple circles, and brown triangles represent magnesium lithospermate B-associated genes, rosmarinic acid-associated genes, and lithospermic acid-associated genes, respectively. Red diamonds represent genes associated with more than one drug. The larger nodes indicate genes with higher degrees. The degree indicates the number of interactions with other proteins in the PPI network.

**Figure 3:**
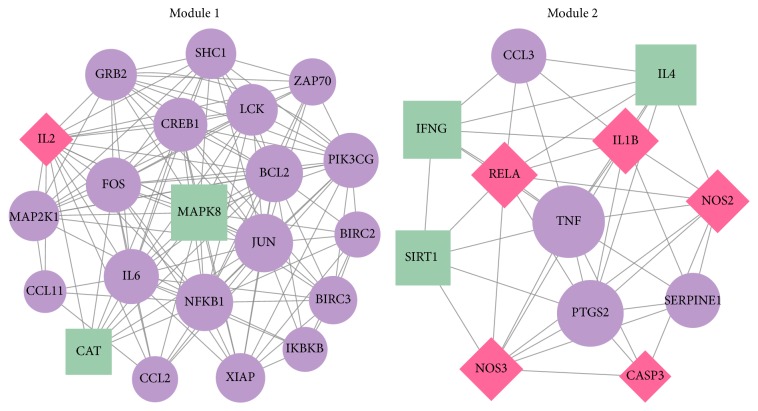
Modules 1 and 2 identified from the protein-protein interaction network. Green squares and purple circles represent magnesium lithospermate B-associated genes and rosmarinic acid-associated genes, respectively. Red diamonds represent genes associated with more than one drug. The larger nodes indicate genes with higher degrees.

**Figure 4:**
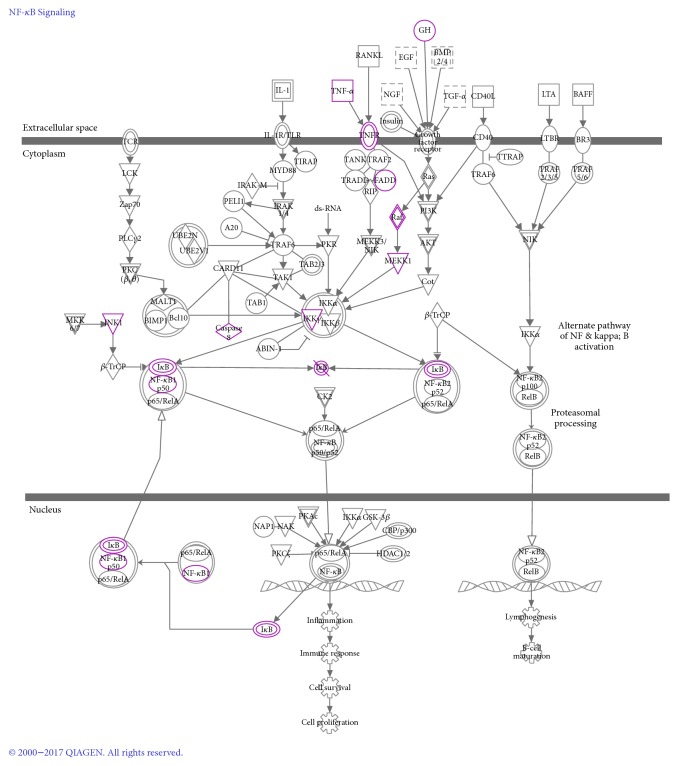
The path diagram of the nuclear factor kappa B (NF-*κ*B) signalling pathway. The highlighted genes represent heart failure-associated genes.

**Figure 5:**
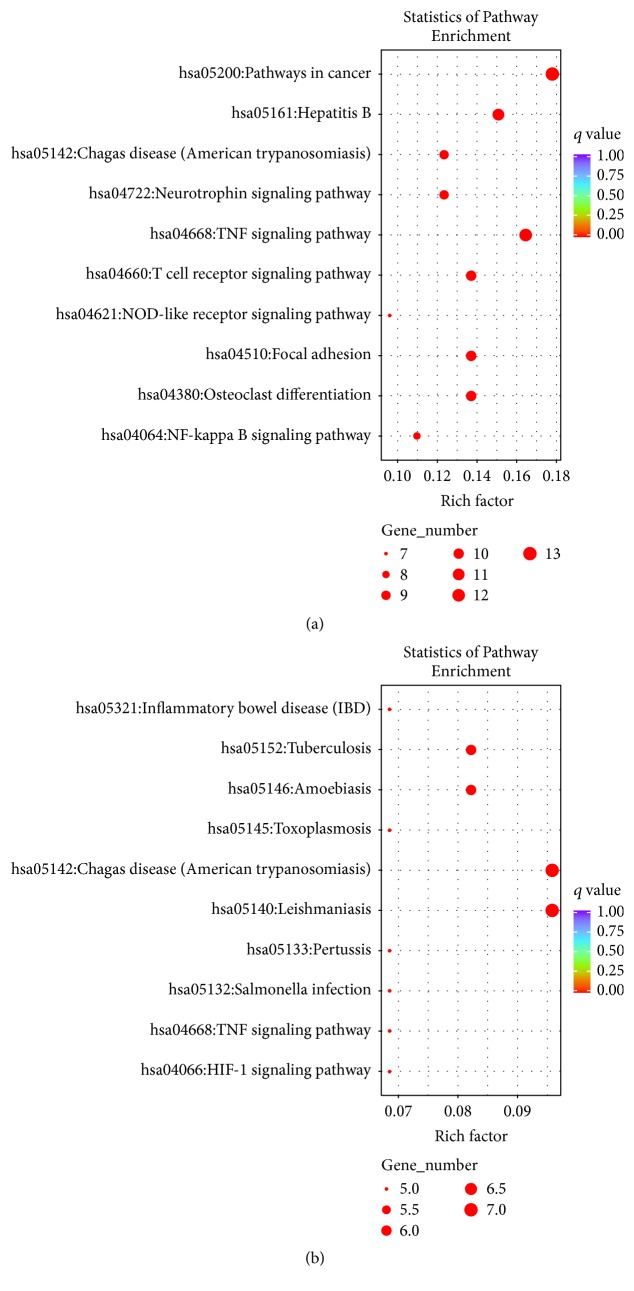
Pathways enriched for the nodes involved in modules 1 (a) and 2 (b).

The PPI network for DSSM-associated genes comprised 65 nodes and 431 edges (Figure 2). The 65 nodes included 21 MLB-associated genes, 56 RA-associated genes, and eight LA-associated genes. Among them, two genes [nitric oxide synthase 2* (NOS2)* and nitric oxide synthase 3* (NOS3)*] were associated with all MLB, RA, and LA; four genes (interleukin 2, interleukin 1 beta, caspase 3, and v-rel avian reticuloendotheliosis viral oncogene homolog A) were associated with both MLB and RA; and four genes (xanthine dehydrogenase, aldo-keto reductase family 1 member B1, protein tyrosine phosphatase no-receptor type 1 and procollagen-lysine, and 2-oxoglutarate 5-dioxygenase) were associated with both MLB and LA. Jun protooncogene (JUN), TNF, nuclear factor kappa B subunit 1 (NFKB1), and Fos protooncogene (FOS) were noted as particular hub nodes according to their BC, closeness centrality, and DC scores (Table 2). Moreover, two modules were also identified from the PPI network: module 1 (21 nodes, score = 13.2) and module 2 (12 nodes, score = 7.8) (Figure 3). In addition, pathway enrichment analysis demonstrated that the nuclear factor kappa B (NF-*κ*B) signalling pathway [Figure 4; *p* = 3.14*E*^−06^; involving* NFKB1* and B-cell CLL/lymphoma 2* (BCL2)*] and leishmaniasis (*p* = 4.72*E*^−07^) were particularly associated with the nodes in modules 1 and 2, respectively (Table 3 and Figure 5). JUN, TNF, NFKB1, FOS, and BCL2 also exhibited notable interactions among themselves in the PPI network.

### 3.4. Transcriptional Regulatory Network Analysis

**Figure 6:**
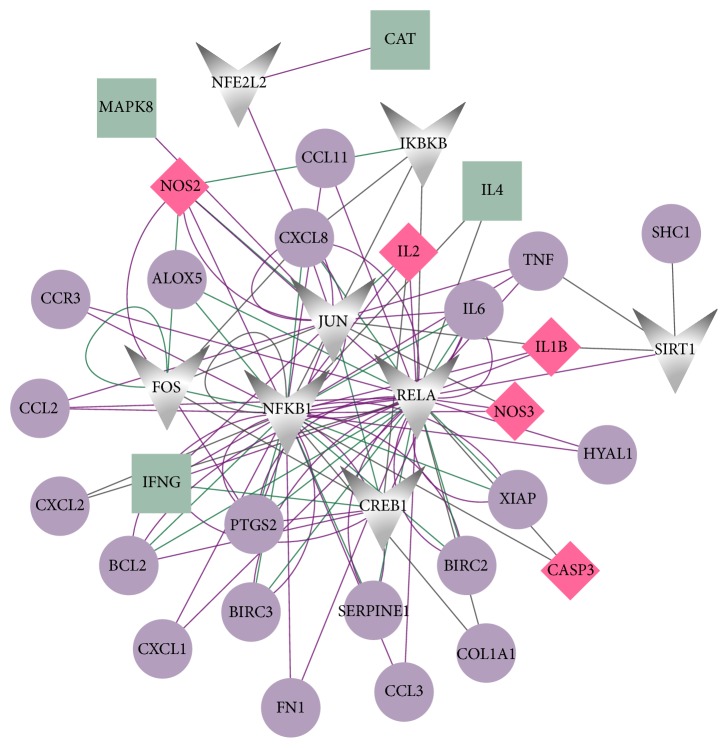
The transcription factor- (TF-) target gene regulatory network. Green squares and purple circles represent magnesium lithospermate B-associated genes and rosmarinic acid-associated genes, respectively. Grey polygons represent TFs. Red diamonds represent genes associated with more than one drug. Larger nodes indicate genes with higher degrees. Purple lines, green lines, and grey lines represent activating regulatory, repressing regulatory, and unknown regulatory effects, respectively.

Based on the TRRUST database, TFs among DSSM-associated genes were searched for and their targets were screened. The constructed transcriptional regulatory network had 37 nodes (including eight TFs and 29 target genes) and 108 relationship pairs (such as* JUN *→* TNF/FOS*,* FOS *→* NFKB1*, and* NFKB1 *→* BCL2/TNF*) (Figure 6).

## 4. Discussion

In this study, a total of 73 DSSM-associated genes were identified from various databases. In the PPI network constructed on the basis of these genes and their interactions, JUN, TNF, NFKB1, and FOS were established as hub nodes according to their BC, closeness centrality, and DC scores. JUN, TNF, NFKB1, FOS, and BCL2 were also shown to interact among themselves in the PPI network. Moreover, two distinct modules (modules 1 and 2) of the PPI network were identified. In addition, several regulatory relationships (such as* JUN *→* TNF/FOS*,* FOS *→* NFKB1*, and* NFKB1 *→* BCL2/TNF*) were found to be involved in the transcriptional regulatory network.

Prolonged anti-TNF-*α* therapy has beneficial effects on the signs of subclinical cardiovascular disease in patients with severe psoriasis [27]. Studies have shown that, compared with nonbiologic disease-modifying antirheumatic drugs, TNF-*α*-blocking agents may contribute to the reduction of the risk of cardiovascular events in patients with rheumatoid arthritis [28, 29]. TNF-*α* plays a critical role in vascular dysfunction in cardiovascular diseases, which may be exploited to treat inflammation in a clinical setting [30]. TNF-*α* boosts atherosclerosis development by promoting the transcytosis of low-density lipoprotein (LDL) across endothelial cells, thus contributing to the reservoir of LDL in vascular walls [31]. These findings indicate that* TNF* may be associated with the functions of DSSM in cardiovascular diseases.

Blocking the JNK pathway may provide a new strategy for treating the cardiomyocyte death induced by myocardial ischaemia/reperfusion [32, 33]. Dominant negative c-Jun* (DN-c-Jun)* gene transfer inhibits VSMC proliferation, and JUN is associated with the intimal hyperplasia induced by balloon injury [34]. Saliques et al. considered that* FOS* is a novel factor that determines the severity and development of atherosclerosis and is thus involved in tobacco toxicity in coronary artery disease (CAD) patients [35]. Palomer et al. also demonstrated that* miR-146a*-targeting* FOS* is a potential tool for treating enhanced inflammation-associated cardiac disorders [36]. Thus,* JUN* and* FOS* may also be targets of DSSM in cardiovascular diseases.

Studies involving Uygur and Han women in China have demonstrated that the DD genotype of* NFKB1* polymorphism (rs28362491) may be a genetic marker of CAD [37, 38]. The NFKB1-94ins/del ATTG polymorphism may reduce the susceptibility to myocardial infarction by decreasing activated NF-*κ*B, which is in turn correlated with the reduction of plasma inflammatory markers [39]. By increasing* BCL2* expression,* miR-21* promotes heart failure progression with preserved left ventricular ejection fraction and subsequently inhibits cardiac fibrosis [40]. Moreover, Tang et al. reported that* miR-1* targets* BCL2* functions in mediating cardiomyocyte apoptosis [41]. In this study, pathway enrichment analysis showing the presence of* NFKB1* and* BCL2* in module 1 indicated a particular association with the NF-*κ*B signalling pathway, indicating that* NFKB1* and* BCL2* may be correlated with the effects of DSSM against cardiovascular diseases through this pathway.

## 5. Conclusion

In conclusion, a total of 73 DSSM-associated genes were identified by conducting a search of public databases.* JUN*,* TNF*,* NFKB1*,* FOS*, and* BCL2* were also revealed as potential targets of DSSM for treating cardiovascular diseases. However, further experimental research should be performed to confirm these findings obtained by bioinformatic analysis.

## Figures and Tables

**Table 1:** MLB-associated genes, RA-associated genes, and LA-associated genes searched from the GeneCards, STICH, SuperTarget, PubChem, and CTD databases.

Database	MLB		RA		LA	
	Count	Symbol	Count	Symbol	Count	Symbol
GeneCards	6	NOS2, NOS3, AKR1B1, XDH, PLOD1, PTPN1	33	SLC16A1, PTGS2, TNF, ALOX5, IL1B, TAT, JUN, BCL2, CXCL8, CCL11, LCK, FOS, CCL2, CREB1, ERVK-6, PIK3CG, NFKB1, PLCG1, IKBKB, SHC1, MAP2K1, CCL3, RELA, GRB2, XIAP, EEF1A1, CCR3, CXCL2, BIRC2, ZAP70, BIRC3, ITK, MIR155	8	NOS2, NOS3, AKR1B1, XDH, PLOD1, PTPN1, SPTLC1, SPTLC2
STICH	8	IL15, IL2, IL4, KCNMA1, KCNU1, MAPK8, RRAGA, RRAGB	7	CCR3, FOS, IKBKB, IL2, LCK, PARG, PROCR	0	/
SuperTarget	0	/	5	ALDR, C1R, HYAL1, LCK, TYRO	0	/
PubChem	0	/	10	PROCR, PARG, TNF, ADAM17, SERPINE1, MAOB, MAOA, IL1B, DNMT1, COL1A1	0	/
CTD	7	IFNG, IL1B, CASP3, NFE2L2, SIRT1, CAT, RELA	21	PROCR, ADAM17, IL1B, CXCL1, IL6, TNF, SERPINE1, AIFM1, CASP3, COL1A1, COMT, DNMT1, FN1, GPT, MAOA, MAOB, NOS2, NOS3, OGG1, PARG, RELA	1	XDH

MLB, magnesium lithospermate B; RA, rosmarinic acid; LA, lithospermic acid, STICH, Search Tool for Interacting Chemicals; CTD, Comparative Toxicogenomics Database.

**Table 2:** Top 10 nodes in the PPI network according to DC, BC, and CC scores.

DC		BC		CC	
Gene	Score	Gene	Score	Gene	Score
JUN	37	CAT	577.2475	JUN	0.186047
TNF	34	FOS	309.3704	TNF	0.184438
NFKB1	34	TNF	256.2388	NFKB1	0.184438
FOS	33	JUN	251.4059	FOS	0.183908
IL6	33	CCL2	205.0727	IL6	0.183381
BCL2	31	NFKB1	186.0261	BCL2	0.181303
MAPK8	29	IL6	185.3155	MAPK8	0.181303
CREB1	28	BCL2	166.0571	CREB1	0.179775
NOS3	25	CREB1	164.3064	NOS3	0.178771
IL1B	24	CASP3	147.608	IL1B	0.178273

PPI, protein-protein interaction; DC, degree centrality; BC, betweenness centrality; CC, closeness centrality.

**Table 3:** Pathways enriched for the genes separately involved in module 1 and module 2 (top 10 listed).

Module	Term	Count	Genes	FDR
Module 1	hsa04668:TNF signaling pathway	12	*PIK3CG, FOS, IL6, CCL2, MAP2K1, JUN, CREB1, NFKB1, MAPK8, BIRC3, IKBKB, BIRC2*	1.18*E* − 12
	hsa05161:Hepatitis B	11	*PIK3CG, FOS, IL6, MAP2K1, GRB2, JUN, BCL2, CREB1, NFKB1, MAPK8, IKBKB*	2.20*E* − 09
	hsa04660:T cell receptor signaling pathway	10	*PIK3CG, FOS, MAP2K1, GRB2, JUN, LCK, ZAP70, NFKB1, IKBKB, IL2*	4.40*E* − 09
	hsa04380:Osteoclast differentiation	10	*PIK3CG, FOS, MAP2K1, GRB2, JUN, CREB1, LCK, NFKB1, MAPK8, IKBKB*	3.98*E* − 08
	hsa05200:Pathways in cancer	13	*PIK3CG, FOS, IL6, XIAP, MAP2K1, GRB2, JUN, BCL2, NFKB1, MAPK8, BIRC3, IKBKB, BIRC2*	9.52*E* − 08
	hsa05142:Chagas disease (American trypanosomiasis)	9	*PIK3CG, FOS, IL6, CCL2, JUN, NFKB1, MAPK8, IKBKB, IL2*	2.56*E* − 07
	hsa04722:Neurotrophin signaling pathway	9	*PIK3CG, MAP2K1, GRB2, JUN, BCL2, NFKB1, MAPK8, SHC1, IKBKB*	8.15*E* − 07
	hsa04510:Focal adhesion	10	*PIK3CG, XIAP, MAP2K1, GRB2, JUN, BCL2, MAPK8, SHC1, BIRC3, BIRC2*	2.33*E* − 06
	hsa04064:NF-kappa B signaling pathway	8	*XIAP, BCL2, LCK, ZAP70, NFKB1, BIRC3, IKBKB, BIRC2*	3.14*E* − 06
	hsa04621:NOD-like receptor signaling pathway	7	*IL6, CCL2, NFKB1, MAPK8, BIRC3, IKBKB, BIRC2*	8.05*E* − 06

Module 2	hsa05140:Leishmaniasis	7	*IL4, TNF, PTGS2, RELA, IFNG, IL1B, NOS2*	4.72*E* − 07
	hsa05142:Chagas disease (American trypanosomiasis)	7	*CCL3, TNF, RELA, SERPINE1, IFNG, IL1B, NOS2*	4.90*E* − 06
	hsa05146:Amoebiasis	6	*CASP3, TNF, RELA, IFNG, IL1B, NOS2*	3.72*E* − 04
	hsa05321:Inflammatory bowel disease (IBD)	5	*IL4, TNF, RELA, IFNG, IL1B*	2.36*E* − 03
	hsa05133:Pertussis	5	*CASP3, TNF, RELA, IL1B, NOS2*	4.47*E* − 03
	hsa05152:Tuberculosis	6	*CASP3, TNF, RELA, IFNG, IL1B, NOS2*	4.76*E* − 03
	hsa05132:Salmonella infection	5	*CCL3, RELA, IFNG, IL1B, NOS2*	6.71*E* − 03
	hsa04066:HIF-1 signaling pathway	5	*RELA, SERPINE1, IFNG, NOS3, NOS2*	1.30*E* − 02
	hsa04668:TNF signaling pathway	5	*CASP3, TNF, PTGS2, RELA, IL1B*	1.78*E* − 02
	hsa05145:Toxoplasmosis	5	*CASP3, TNF, RELA, IFNG, NOS2*	2.72*E* − 02
